# UFGT: The Key Enzyme Associated with the Petals Variegation in Japanese Apricot

**DOI:** 10.3389/fpls.2017.00108

**Published:** 2017-02-07

**Authors:** Xinxin Wu, Qinghua Gong, Xiaopeng Ni, Yong Zhou, Zhihong Gao

**Affiliations:** ^1^Laboratory of Fruit Tree Biotechnology, College of Horticulture, Nanjing Agricultural UniversityNanjing, China; ^2^Jiangsu Key Laboratory for Horticultural Crop Genetic ImprovementNanjing, China; ^3^The Administration Bureau of Sun Yat-sen's MausoleumNanjing, China

**Keywords:** anthocyanins content, enzyme activity, Japanese apricot, RNA-seq, transcriptome analysis, UFGT, variegation, nonsynonymous mutation

## Abstract

Japanese apricot (*Prunus mume* Sieb.et Zucc.) is an important ornamental plant in China. One of the traits of petals color variegation is attractive, but its formation mechanism is unclear. In our study, RNA-seq technology was employed to characterize the transcriptome response to the mutation of “Fuban Tiaozhi” associated with petals variegation in Japanese apricot. As a result, 4,579,040 (white-flowered, WF) and 7,269,883 (red-flowered, RF) reads were mapped to *P. persica* genes, while 5,006,676 (WF) and 7,907,436 (RF) were mapped to *P. persica* genomes. There were 960 differentially expressed genes (DEGs) identified. Gene ontology analysis showed that these genes involved in 37 functional groups including 19 biological processes, 10 cellular components and eight molecular functions. Pathway enrichment annotation demonstrated that highly ranked genes were associated with flavonoid biosynthesis, anthocyanin biosynthesis, anthocyanins transports, plant hormone signal transduction, and transcriptional factors. The expression patterns part of them were validated by qRT-PCR. We found that UDP-glucose: flavonoid 3-*O*-glucosyltransferase (*UFGT*) gene showed differential expression pattern. The UFGT enzyme activities in RF had a significantly higher than that of WF and lower in the initial stage and increased when the red appeared in the petals, which is identical to the accumulation of anthocyanins. And we also validated the SNPs, leading to the nonsynonymous mutations, in the *UFGT* by Sanger sequencing which may affect the enzyme activity. In summary, our results provide molecular candidates for better understanding the mechanisms of the variegation in Japanese Apricot.

## Introduction

Japanese apricot (*Prunus mume* Siebold & Zucc.), belongs to the *Rosaceae*, subfamily Prunoideae, genus *Prunus* L., is diploid (2*n* = 16). It originated in Southwestern China and has been domesticated in China for more than 7000 years (Sun et al., 2013). Japanese apricot is now widely cultivated as an early-blooming garden ornamental plant in East Asian countries, because of its rich colors and outstanding ornamental characteristics (Chu, 1999). Despite its importance, we have little knowledge about the genetic mechanisms that underlie biological and ornamental traits of Japanese apricot.

Flower color is important for attracting pollinators and is a critical factor in plant survival. In addition, the color of the flower is a central trait of ornamental plants and is determined by plant pigments, including flavonoids, carotenoids, and betalains (Tanaka et al., 2008). Various patterns in flower color are observed in nature. Variegated plants, different colored petals on the same tree, are valuable in the floricultural market because they often attract consumer attention. This phenotype has been observed in *Petunia, Snapdragon, Prunus persica*, and other plant species (Wang et al., 2015; Zhou et al., 2015a). The formation of the flower color is a complex progress that regulated by lots of gene. There are two categories of genes, structural and regulatory genes, involved in anthocyanin biosynthesis (Wei et al., 2015). The first are structural genes encoding enzymes that catalyze each step of the biosynthetic pathway. Genetic engineering of flower has been reported in recent literatures, e.g., chalcone synthase (CHS), flavonoid 3′-hydroxylase (F3′H), leucoanthocyanin dioxygenase/anthocyanin synthase (LDOX/ANS) (Tanaka et al., 2009; Zhao et al., 2016). CHS is the first committed enzyme in the flavonoid biosynthetic pathway, F3′H and F3′5′H are the direct enzymes for synthesizing colored anthocyanins (Forkmann and Martens, 2001). LDOX/ANS catalyzes leucoanthocyanidins into anthocyanidins. Anthocyanins further glycosylated by uridine diphosphate UDP-glucose: flavonoid-*O*-glycosyltransferase (UFGT) (Chen et al., 2012). The glycosylation and methylation of anthocyanins causes the color to become slightly redder (Tanaka et al., 2010). The second types are regulatory genes encoding MYB transcription factors (TFs), WD repeat proteins and basic helix-loop-helix (bHLH) TFs (Lin-Wang et al., 2010; Saito et al., 2013). These TFs and the complex mediate the regulation of the anthocyanin biosynthetic pathway (Xu et al., 2015). Other factors contributing to flower coloration include pigment structure and concentration, co-pigments, vascular pH, metal ion type, cell shape, and plant hormones signal.

Plant hormones, such as cytokinins, abscisic acid (ABA), jasmonates (JAs), and ethylene (ET), are important signaling molecules in response to biotic stresses and flower color (Jaakola, 2013). It also affected the anthocyanin biosynthesis by interacting with the MYB-bHLH-WD40 complexes at transcriptional or the post-transcriptional level. Cytokinins have been found to induce anthocyanin biosynthesis in *Arabidopsis* (Das et al., 2012). JAs affect color formation via interaction with ET biosynthesis in plants (Rudell et al., 2005; Mizuno et al., 2016). In *Arabidopsis*, JAs affect anthocyanins accumulation via interaction of JA ZIM domain proteins with MYB-bHLH-WD40 complex (Qi et al., 2011). In grape, a fully functional pathway for ET synthesis activated just before red berries start to accumulate anthocyanins (Chervin et al., 2004). In *Cymbidium hybrida*, the *ChMYB1* is shown to be an MYB related to anthocyanins and control the anthocyanins production mediated by ET signaling (Lewis et al., 2015). And the exogenous ET could induce structural gene *ufgt* expression (Chervin et al., 2009). Anthocyanin accumulation is influenced by the plant hormone ABA during the veraison stage in grape berries (Ferrara et al., 2015).

RNA-seq is now regarded as the most powerful tool for sequencing and profiling of transcriptome, because it has high sensitivity and great base-pair resolution. It requires less prior knowledge of gene sequences and it can detect a larger range of expression values than other methods (Marioni et al., 2008). In the last several years, RNA-seq has become the platform of choice for sequencing and profiling transcriptome and has been widely used, e.g., *Prunus* (Martínez-Gómez et al., 2011), *Pyrus* (Liu et al., 2012), *Cymbidium sinense* (Zhang et al., 2013). To facilitate isolation of genes controlling important horticultural traits of peach, deep RNA-seq were used to uncover the peach transcriptome landscape (Wang et al., 2013). In addition, the technology can be used not only for analysis of static genomes, but also to analyze dynamic transcriptome (Huang et al., 2014). Zhong et al. (2013) applied Illumina sequencing to reveal the comprehensive mechanism of seasonal bud dormancy at four critical stages in Japanese apricot at the transcriptional level. There were 6199, 5539, and 5317 DEGs in R1 vs. R2, R2 vs. R3, and R3 vs. R4, respectively. Rubio et al. (2015) detected differences in gene expression after *Plum pox virus* infection in peach GF305 leaves with and without *sharka* symptoms using RNA-seq. In the study of Chen et al. (2014) they identified candidate genes associated with variegation in peach flowers, including C4H, CHS, CHI, and F3H.

Detection of differentially expressed genes (DEGs) from both two colored flowers is an essential step to elucidate mechanisms of variegated pigmentation. Here, we used the next-generation high-throughput sequencing technology to investigate these mechanisms. DEGs were identified as candidate genes related to variegated pigmentation at the transcriptional level in Japanese apricot. Our data presented here may be a useful resource for further study.

## Materials and methods

### Plant materials

**Figure 1:**
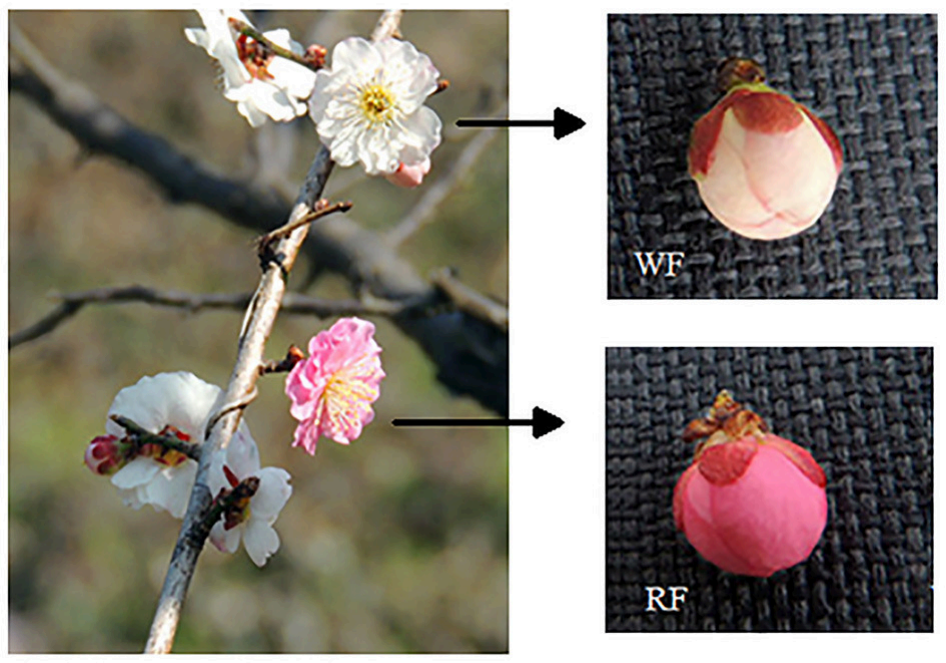
**Examples of white (WF) and red (RF) buds in the pink stage from trees grown at Meihua Hill, Ming Dynasty Xiaoling Tomb, Nanjing, Jiangsu Province, China**.

This study used trees of the Japanese apricot cv “Fuban Tiaozhi” Mei grown at Meihua Hill, Ming Dynasty Xiaoling Tomb, Nanjing, Jiangsu Province, China in March 2014. “Fuban Tiaozhi” Mei can display both white (light red stain with white background) and red flowers on different branches of the same plant, and different colors can be within a single petal. We separately collected flower buds with white (WF) and red (RF) petals from three stages: 0 day is the initial stage (small buds and petal color unobserved); 7 day is the red stage (RF buds with red point and WF buds with white or variegated point at the top); and 14 day is the balloon stage (petals color easily observed). Seven day samples were taken for RNA isolation and RNA-seq analysis (Figure 1). Each collection was performed with three biological replicates. RNAs isolated from the three replicates were mixed at 1:1:1 ratio for library construction and sequencing. Three stages samples were used for analyzing UFGT enzyme activity with three biological replicates. Both WF and RF petals were collected from newly opened flowers for analyzing the content of anthocyanins. All samples were immediately frozen in liquid nitrogen and stored at −70°C until use.

### Anthocyanin determination

Anthocyanin concentrations were determined using the pH differential method (Wrolstad et al., 1982; Zhou et al., 2015b), with a spectrophotometer (UV-2450, Shimadzu Corporation, Japan). Approximately 0.1 g petal flour was added to 10 ml 1% HCl. Pigment extracts were made up to a volume to 25 ml. Pigment extract (2 ml) was mixed with 3 ml 0.4 M KCl-HCl buffer (*pH* = 1) and a separate 2 ml was mixed with 3 ml 0.4 M Na_2_HPO_4_-Citric acid buffer (*pH* = 5). Absorbance was measured at 510 nm. Measurements of anthocyanin were replicated three times.

### RNA extraction and sequencing

Total RNA was isolated from white and red flower buds and the libraries were named WF and RF, respectively. RNA extraction was performed by the manufacturer's instructions using Trizol reagent (Invitrogen, USA). The RNA concentration and quality were detected by UV spectrophotometry and by running on a 1.2% agar/ethidium bromide gel. The total RNA samples were first treated with DNase I (TaKaRa, Dalian, China) to degrade DNA contamination. The mRNA was enriched using oligo (dT) magnetic beads then fragmented to approximately 200 bp by mixing with fragmentation buffer. Then, random hexamer-primer was used to synthesize the first-strand cDNA using the cDNA Synthesis Kit (Stratagene, Cedar Creek, USA) following the manufacturer's instructions. The short fragments were purified using the QiaQuick PCR extraction kit (Qiagen, Valencia, CA) to repair the end by adding a poly(A) tail. Sequencing adaptors were ligated to the fragments. The fragments were amplified by PCR. During the QC step, an Agilent 2100 Bioanaylzer and the ABI StepOnePlus Real-Time PCR System were used to qualify and quantify the sample libraries. The library products were prepared for sequencing via Illumina HiSeq™ 2000 following the manufacturer's protocols.

### Treatment of sequence data and reads mapping

The original image data produced by the sequencer was transferred into sequences by base calling. As raw reads may include low quality reads and or adaptor sequences, pre-processing was necessary before starting further analysis. Data were filtered to obtain high quality clean reads. Clean reads were mapped to reference sequences and reference gene sets using a SOAPaligner/SOAP2. No more than two mismatches were allowed in the alignment. The frequency of occurrence of individual reads was normalized to RPKM (reads per kb per million reads). Sequences were compared using Blastn and the following database: the national center for biotechnology information (NCBI), the peach [*Prunus persica* (L.) Batsch] genome database (https://www.rosaceae.org/species/prunus_persica/genome_v1.0; Verde et al., 2013). Unigene annotation was against peach protein (http://www.phytozome.org). The raw sequence data sets were deposited in the NCBI Sequence Read Archive (SRA, http://www.ncbi.nlm.nih.gov/Traces/sra; Wheeler et al., 2008) under accession number SRR5124653 (WF) and SRR5124917 (RF).

### Screening of DEGs

To identify the DEGs between the two libraries, the number of raw clean tags for each library was normalized against the number of RPKM to obtain the normalized gene expression level. The DEGs were identified using the method described by Audic and Claverie (1997). We used FDR ≤ 0.001 and the absolute value of log_2_ ratio (RF/WF) ≥ 1 as the threshold to judge the significance of gene expression difference.

To analyze the main biological functions, we mapped all DEGs to the terms in the GO (gene ontology) database (http://www.geneontology.org/) and KEGG (Kyoto encyclopedia of genes and genomes; (Kanehisa et al., 2008)). We chose a corrected *P* < 0.05 as a significantly DEGs-enriched GO term. This analysis is able to recognize the main biological functions of DEGs. We used the Blast2GO program (version: v2.5.0) to obtain GO annotation of DEGs. We then used WEGO software (http://wego.genomics.org.cn/cgi-bin/wego/index.pl) to do GO functional classification for DEGs and to understand the distribution of gene functions of the species at a macro level.

Genes usually interact with each other to play certain roles in biological functions. Pathway-based analysis helps to further understand the biological functions of genes. KEGG is the major pathway-related database, and assigns functions based on the Enzyme Commission (EC). The calculating formula is the same as that in GO analysis. The corrected *Q* < 0.05 was chosen as the threshold value to identify significantly DEGs in the pathway analysis.

### Real-time quantitative RT-PCR analysis

The expression of candidate genes was verified using qRT-PCR. The same RNA samples were used for the qRT-PCR assays as well as for the RNA-seq experiments. First-strand cDNA was synthesized from total RNA using Superscript II reverse transcriptase (Invitrogen, San Diego, CA). Gene-specific primers were designed according to gene sequences using Beacon Designer 7 program (Premier Biosoft, Palo Alto CA). All primers are listed in Supplemental Table 1. Primers specific for *actin* were used to normalize the reactions (Tong et al., 2009). Experiments were performed using the method described by Zhong et al. (2013). The following thermal cycling profile was used: 95°C for 3 min, 40 cycles at 95°C for 25 s, 62°C for 25 s, and 72°C for 40 s. Transcript abundances are given as the mean ± SE of three replicates. The relative expression level of the genes was calculated using the 2^−ΔCTΔCT^ method.

### UFGT enzyme activity

To obtain functional enzymes, 1 g *P. mume* buds was frozen in liquid nitrogen and ground to a fine powder with a mortar and pestle. The powder was then extracted with 7.5 ml boric acid extraction buffer (pH 8.8, including 5 mM β-mercaptoethanol, 1 mM EDTA, and 1 mM DTT) and 10% PVPP, homogenized in ice bath. The mixture was then centrifuged at 12,000 g, 4°C for 20 min, and supernatant was used for enzyme assays. The reaction solution contained 100 μl enzyme supernatant, 100 μl 50 mM glycine buffer (*ph* = 8.5), 15 μl 2 mM quercetin (Sigma Chemical, St. Louis, USA) and 10 μl 15 mM UDP-galactose (Sigma Chemical, St. Louis, USA). Reaction tubes were incubated at 30°C for 30 min and the reaction terminated by the addition of 20% trichloro acetic acid in methanol (75 ul). The homogenate was centrifuged at 6000 × g for 5 min at 4°C. The supernatant of each solution was filtered through 0.45 Millipore filter, and stored at −20°C until quantification of enzyme by Ultra Performance Liquid Chromatography (UPLC).

UPLC (ACQUITY UPLC H-Class Core System, Waters, Inc., USA) was equipped with a reverse phase column ACQUITY UPLC HSS C18 (1.8 μm particle sizes, 100 × 2.1 mm I.D., Waters, USA) for separation. The UPLC method was according to Lister et al. (1996) with some modification in total elution time. The mobile phase was water: acetic acid (99.5:0.5) as solvent A, and acetonitrile as solvent B. The gradient profile began at 95% A at 2.8 min, 45% A at 3.5 min, 40% A at 4 min, and then returned to initial conditions at 4 min and for 1 min. The flow rate was 0.35 ml/min and the column temperature was set at 30°C. The injection volume was 2 μl in the Waters system. Anthocyanins were detected by UV absorbance at 360 nm in order to obtain chromatograms. UFGT was quantified using quercetin-3-galactoside (Sigma chemical, St. Louis, USA) as a standard. Finally, the UFGT activity was represented with: mg quercetin-3-gal·g-1 FW. Each sample was injected thrice for biological replication.

### Sanger sequencing to identification nonsynonymous mutation in UFGT

One single nucleotide polymorphism (SNP) related to UFGT was chosen, which induce amino acid changes in the CDS region, was subjected to validation using PCR and Sanger sequencing with WF and RF samples. Genomic DNA was isolated from the samples using the CTAB method following manufacturer's instructions. Isolated DNA was quantified using Onedrop OD-1000+ spectrophotometer (Onedrop, Shanghai, China) and a dilution of 1 ng/μl was prepared in 96 well PCR format for all samples. For PCR reaction, forward and reverse primers were designed according to UFGT 3 gene sequence (NCBI gene ID: 103328988) using Oligo version 7.60 (Molecular Biology Insights, Inc., USA; Supplemental Table 1). The sequencing primers were designed to anneal at least 50 bp upstream of the SNP position and forward/reverse primers were chosen at the flanking regions of the sequencing primer and the SNP position. All primers were commercially synthesized by TSINGKE Biological Technology (Nanjing, China). PCR was carried out as 25 ul reaction volumes in 96 well plates with cycle conditions as follows: denaturation 95°C for 1 min, 35 cycles of amplification (95°C for 30 s, 50°C for 30 s, 72°C for 40 s), final extension 72°C for 10 min. Verification of PCR was performed by 1% agarose gel electrophoresis. Purified PCR products were subjected to Sanger sequencing performed by TSINGKE Biological Technology (Nanjing, China). Results were analyzed using chromas 2 software (Technelysium Pty Ltd, Helensvale, Australia).

## Results

### Anthocyanin contents

**Figure 2:**
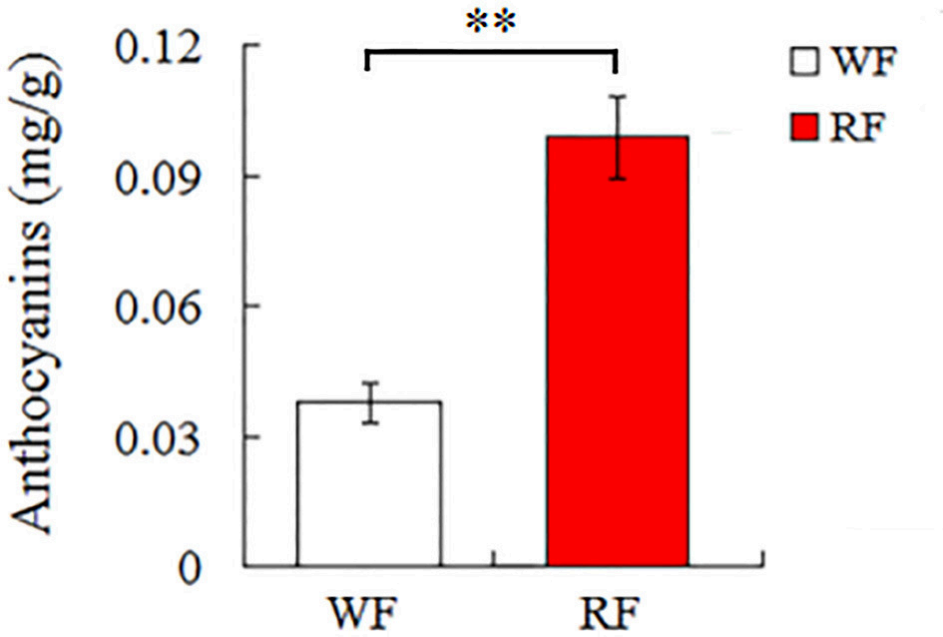
**Anthocyanin content of white (WF) and red (RF) flowers samples in Japanese apricot**. Asterisk indicates significant differences (^**^p < 0.05).

The anthocyanin content in red petals was higher (0.099 mg/g) than that of white petals (0.038 mg/g) with the pH differential method (Figure 2). The anthocyanin is the main pigment in petals that affect the color appearance.

### Analysis of RNA-seq libraries

After filtering out dirty tags from the raw data, the number of clean tags was 11,669,841 (RF) and 11,983,115 (WF) and the total number of base pairs was 571,822,209 (RF) and 587,172,635 (WF) in Table 1. The number of discarded sequences was 86,776 (0.74%, RF) and 91,986 (0.76%, WF; Supplemental Table 2). There were 4.58 and 7.27 million tags mapped to reference genes and 5.01 and 7.91 million tags mapped to the reference genome.

**Table 1:** **Statistics of RNA-seq libraries of red (RF) and white (WF) flowers of Japanese apricot mapped to gene and genome**.

**Sample ID**		**Total reads**	**Total base pairs**	**Total mapped reads**	**Perfect match**	**≤ 2 bp mismatch**	**Unique match**	**Multi-position match**	**Total unmapped reads**
RF	Map to Gene	11,669,841 (100.00%)	571,822,209 (100.00%)	4,579,040 (39.24%)	2,113,194 (18.11%)	2,465,846 (21.13%)	4,079,271 (34.96%)	499,769 (4.28%)	7,090,801 (60.76%)
	Map to Genome	11,669,841 (100.00%)	571,822,209 (100.00%)	5,006,676 (42.90%)	2,097,261 (17.97%)	2,909,415 (24.93%)	4,577,694 (39.23%)	428,982 (3.68%)	6,663,165 (57.10%)
WF	Map to Gene	11,983,115 (100.00%)	587,172,635 (100.00%)	7,269,883 (60.67%)	3,323,921 (27.74%)	3,945,962 (32.93%)	6,469,765 (53.99%)	800,118 (6.68%)	4,713,232 (39.33)
	Map to Genome	11,983,115 (100.00%)	587,172,635 (100.00%)	7,907,436 (65.99%)	3,228,558 (26.94%)	4,678,878 (39.05%)	7,300,191 (60.92%)	607,245 (5.07%)	4,075,679 (34.01%)

### Real-time RT-PCR analysis

**Figure 3:**
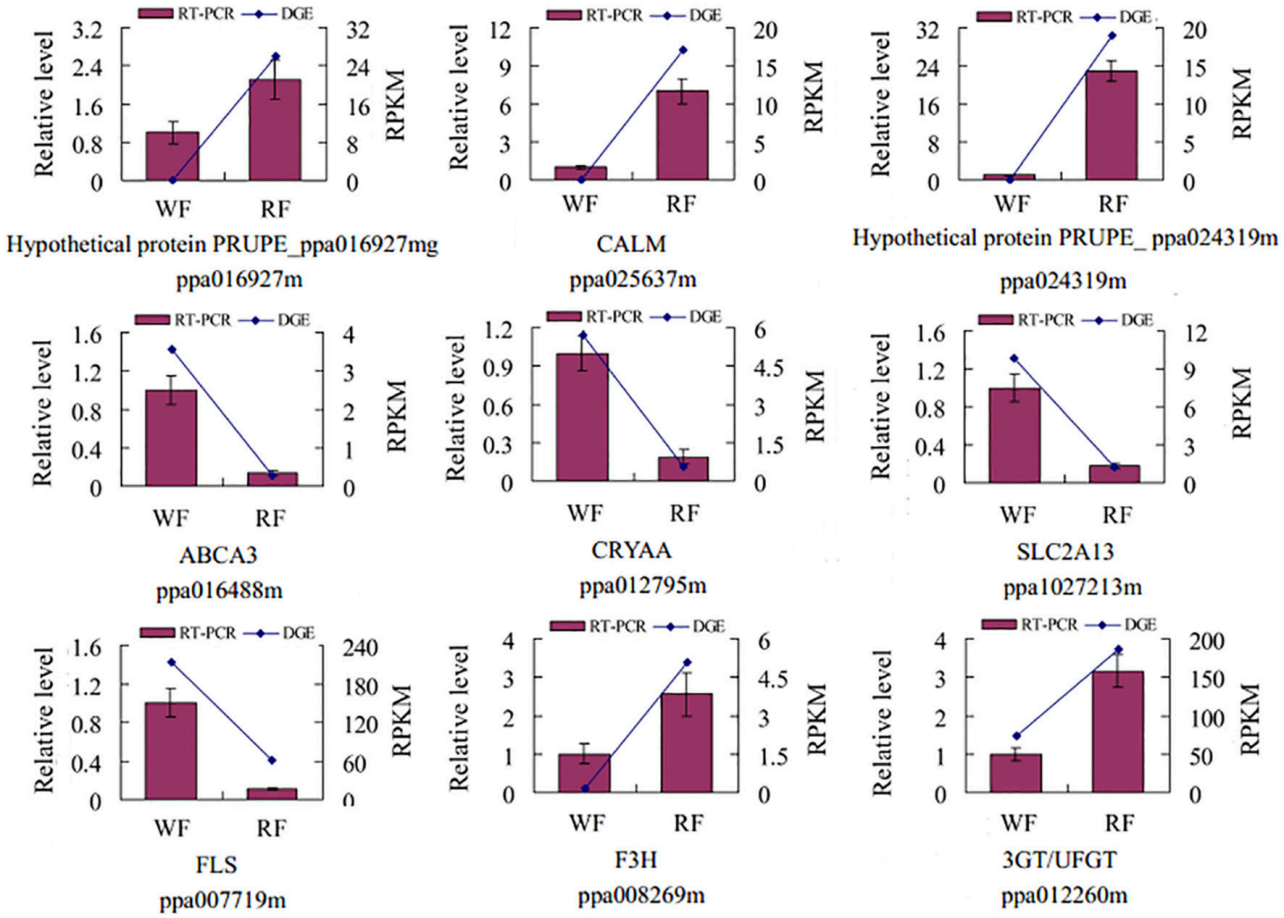
**Validation of the tag-mapped genes with quantitative RT-PCR in Japanese apricot**. The x-axis indicates the two libraries. The y-axis shows the gene expression levels: the left side is the relative expression level as determined by quantitative RT-PCR (red shaded bars), whereas the right side is reads per kb per million reads by RNA-seq (blue line). Calmodulin (*CALM*), MFS transporter, SP family, solute carrier family 2 member 13 (*SLC2A13*), anthocyanidin/flavonol 3-*O*-glucosyltransferase (*3GT/UFGT*), crystallin, alpha A (*CRYAA*), ATP-binding cassette, subfamily A member 3 (*ABCA3*), naringenin 3-dioxygenase (*F3H*), flavonol synthase (*FLS*).

To validate the reliability of the RNA-seq, nine genes were randomly selected for qRT-PCR analysis (Figure 3). The expression of each DEG in the differently colored samples was compared with its abundance from the sequencing data from RNA-seq. The relative expression levels of the genes were calculated in qRT-PCR analysis. The result indicated good reproducibility between the RNA-seq and the qRT-PCR.

### GO functional classification of DEGs

**Figure 4:**
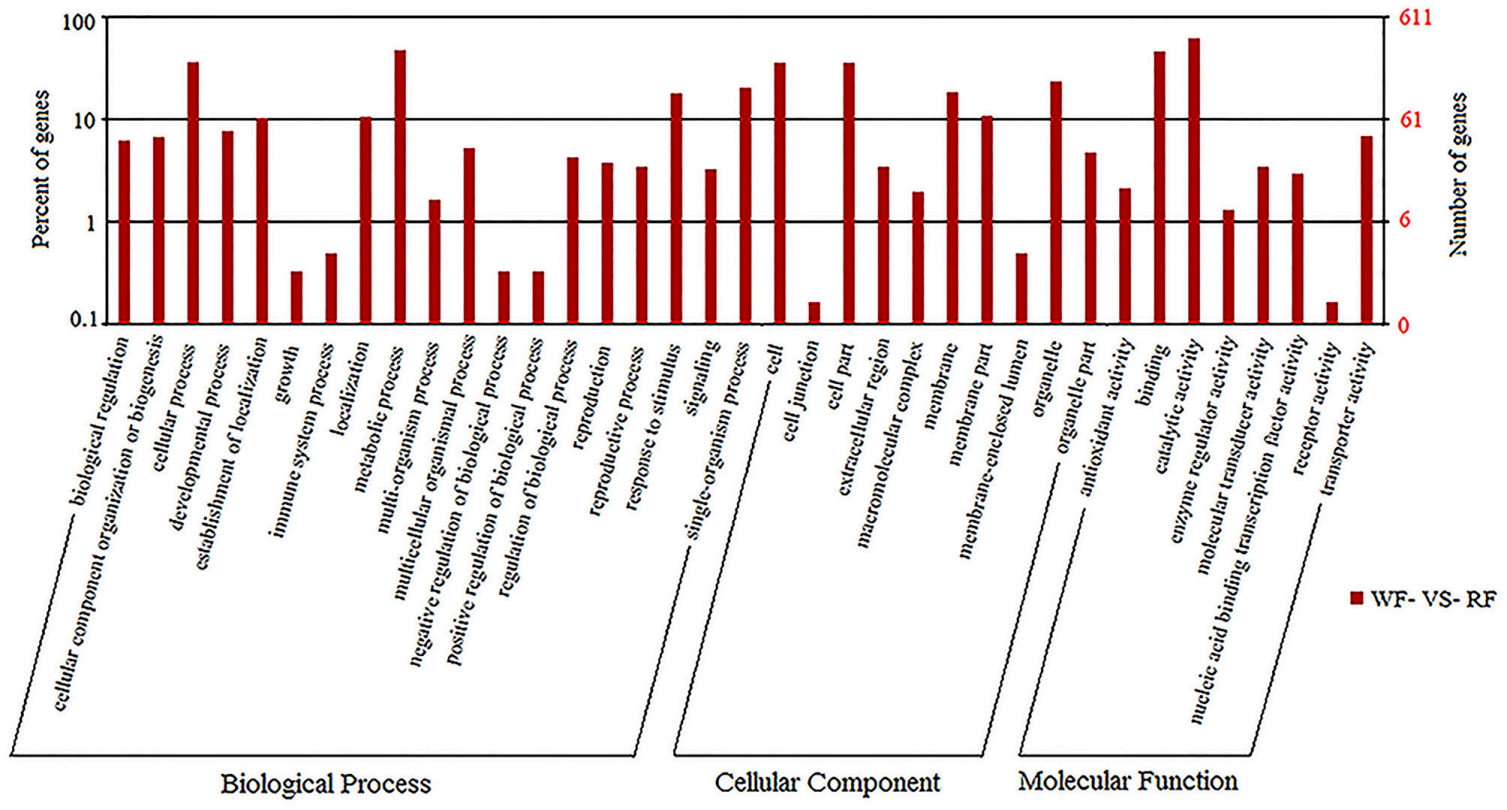
**The GO frequencies and the numbers of gene in each term were shown in histograms**.

GO covers three domains: biological processes, molecular functions and cellular components. The *P* ≤ 0.05 as the threshold value was used to analyze the major biological functions of DEGs. The comparison of WF vs. RF identified 37 functional groups, which included 19 biological processes, eight molecular functions and 10 cellular components (Figure 4). The biological processes identified involved cellular processes, metabolic processes, responsive to stimulus and single-organism processes. More genes were assigned to the biological process than cellular component and the molecular function. A significant number of cellular component GO terms were associated with cell, cell part, organelle and membrane functions. In molecular functions categories, a large number of genes were involved in catalytic activity and binding.

### Pathway analysis

**Figure 5:**
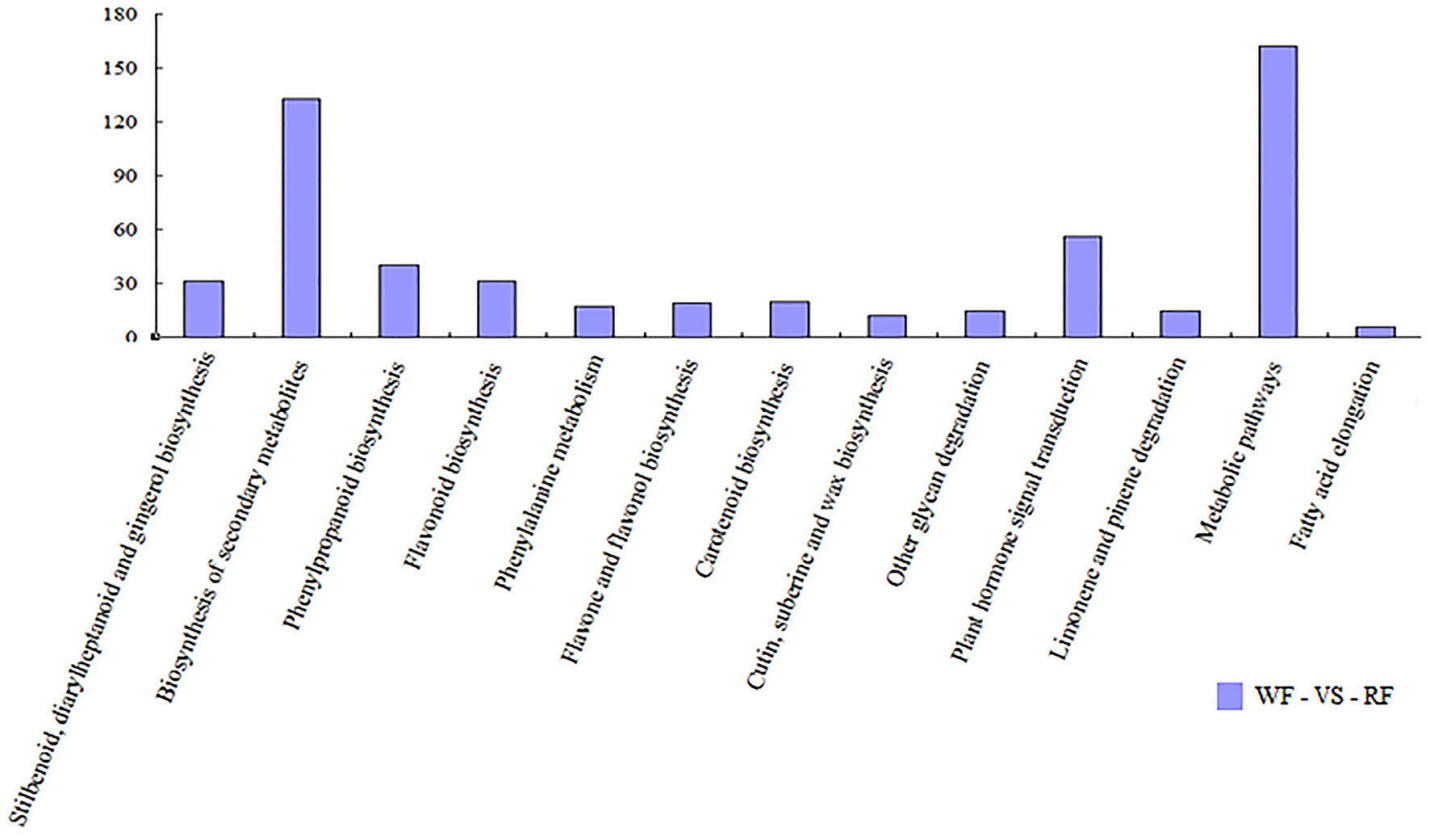
**Histogram showing pathway categories and the numbers of gene in each pathway, respectively**.

We used pathway analysis of the DEGs to understand further the biological functions of DEGs associated with flower color, based on the KEGG database. We selected the significant DEGs with a *Q* ≤ 0.05 as pathway enrichment (Figure 5). Flavonoids (flavonoid biosynthesis, ko00941, 31 genes) are plant secondary metabolites that have a wide variety of functions including pigmentation and antioxidant activity (Agati et al., 2012). They were synthesized from phenylpropanoid derivatives by condensation with malonyl-CoA. Flavone and flavonol biosynthesis (ko00944, 19 genes) and anthocyanin biosynthesis (ko00942, 3 genes) are part of the flavonoid modification pathways. By comparing the two libraries, 53 differentially transcribed genes were found in three pathways involving flavonoids (Table 2). Plant hormone signal transduction (ko04075, 56 genes) may have contributed to phenotypic changes (Mu et al., 2012). Annotations of DEGs were identified in the peach genome annotation, and most of the genes were assigned to a Pfam category.

**Table 2:** **The DEGs involved in flavonoid biosynthesis**.

**Gene ID**	**WF-RPKM**	**RF-RPKM**	**Log_2_ Ratio (RF/WF)**	**Gene annotation**
**WF VS. RF UP-REGULATING**				
ppa024512m	0.49	10.26	4.40	Leucoanthocyanidin reductase (LAR)
ppa014940m	0.75	8.52	3.51	Naringenin 3-dioxygenase (F3H)
ppa004937m	0.32	3.54	3.47	Shikimate O-hydroxycinnamoyltransferase (HCT)
ppa004436m	2.52	19.21	2.93	Flavonoid 3′-monooxygenase (F3′H)
ppa022132m	0.63	4.33	2.78	Flavonoid 3′-monooxygenase (F3′H)
ppa008269m	1.97	13.48	2.77	Naringenin 3-dioxygenase (F3H)
ppa008603m	1.36	7.20	2.40	Polyketide reductase (PKR)
ppa005318m	15.52	79.31	2.35	Shikimate O-hydroxycinnamoyltransferase (HCT)
ppa021776m	1.59	7.32	2.21	Flavonol 3-*O*-methyltransferase (3-OMT)
ppa018282m	1.64	7.30	2.15	Trans-cinnamate 4-monooxygenase (C4H)
ppa019829m	3.95	15.85	2.00	Leucoanthocyanidin reductase (LAR)
ppa004639m	2.08	7.74	1.90	Flavonoid 3′-monooxygenase (F3′H)
ppa007511m	2.20	7.76	1.82	Flavonol 3-*O-*methyltransferase (3-OMT)
ppa008529m	3.92	12.19	1.64	Polyketide reductase (PKR)
ppa007742m	3.17	9.14	1.53	Flavonol 3-*O*-methyltransferase (3-OMT)
ppa022307m	54.24	145.48	1.42	Shikimate O-hydroxycinnamoyltransferase (HCT)
ppa017446m	16.16	42.36	1.39	Leucoanthocyanidin reductase (LAR)
ppa004474m	67.74	176.53	1.38	Coumaroylquinate (coumaroylshikimate) 3′-monooxygenase
ppa005162m	106.96	272.14	1.35	Anthocyanidin/Flavonol 3-*O*-glucosyltransferase (3GT/UFGT)
ppa012260m	74.03	185.78	1.33	Anthocyanidin/Flavonol 3-*O*-glucosyltransferase (3GT/UFGT)
ppa004473m	71.89	175.73	1.29	Coumaroylquinate (coumaroylshikimate) 3′-monooxygenase
ppa004544m	127.91	303.00	1.24	Trans-cinnamate 4-monooxygenase (C4H)
ppa005910m	15.04	34.66	1.20	Shikimate O-hydroxycinnamoyltransferase (HCT)
ppa016640m	71.60	162.48	1.18	Shikimate O-hydroxycinnamoyltransferase (HCT)
ppa004095m	9.54	21.18	1.15	Flavonoid 3′-monooxygenase (F3′H)
ppa015221m	49.65	109.32	1.14	Anthocyanidin/Flavonol 3-*O*-glucosyltransferase (3GT/UFGT)
ppa007994m	24.40	49.11	1.01	Leucoanthocyanidin reductase (LAR)
ppa004404m	13.02	32.22	1.31	Coumaroylquinate (coumaroylshikimate) 3′-monooxygenase
ppa004406m	5.54	11.81	1.09	Coumaroylquinate (coumaroylshikimate) 3′-monooxygenase
**WF VS. RF DOWN-REGULATING**				
ppa017300m	14.91	1.88	−2.99	Flavonoid 3′-monooxygenase (F3′H)
ppa019432m	7.56	1.13	−2.74	Chalcone synthase (CHS)
ppa027160m	36.72	6.73	−2.45	Flavonoid 3′-monooxygenase (F3′H)
ppa023872m	109.29	21.95	−2.32	Leucoanthocyanidin reductase (LAR)
ppa019048m	7.29	1.77	−2.05	Flavonoid 3′-monooxygenase (F3′H)
ppa007719m	213.00	61.33	−1.80	Leucoanthocyanidin dioxygenase/ Flavonol synthase
ppa005715m	27.66	8.95	−1.63	Shikimate O-hydroxycinnamoyltransferase (HCT)
ppa015896m	32.96	16.17	−1.03	Flavonol 3-*O*-methyltransferase (3-OMT)
ppa005581m	108.72	54.26	−1.00	Shikimate O-hydroxycinnamoyltransferase (HCT)

### Variation in transcription level between WF and RF libraries

**Figure 6:**
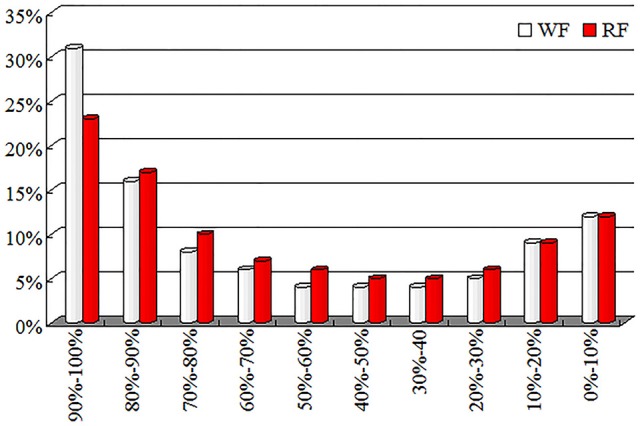
**Distribution of gene coverage for WF and red RF flowers of Japanese apricot**.

The distribution of unigenes coverage in each sample was analyzed as a way of evaluating the quality of the RNA-seq dataset (Figure 6). The proportion of the full gene sequence represented by RNA-seq reads were reflected by gene coverage. Most of the unigenes, gene coverage was >50%.

**Figure 7:**
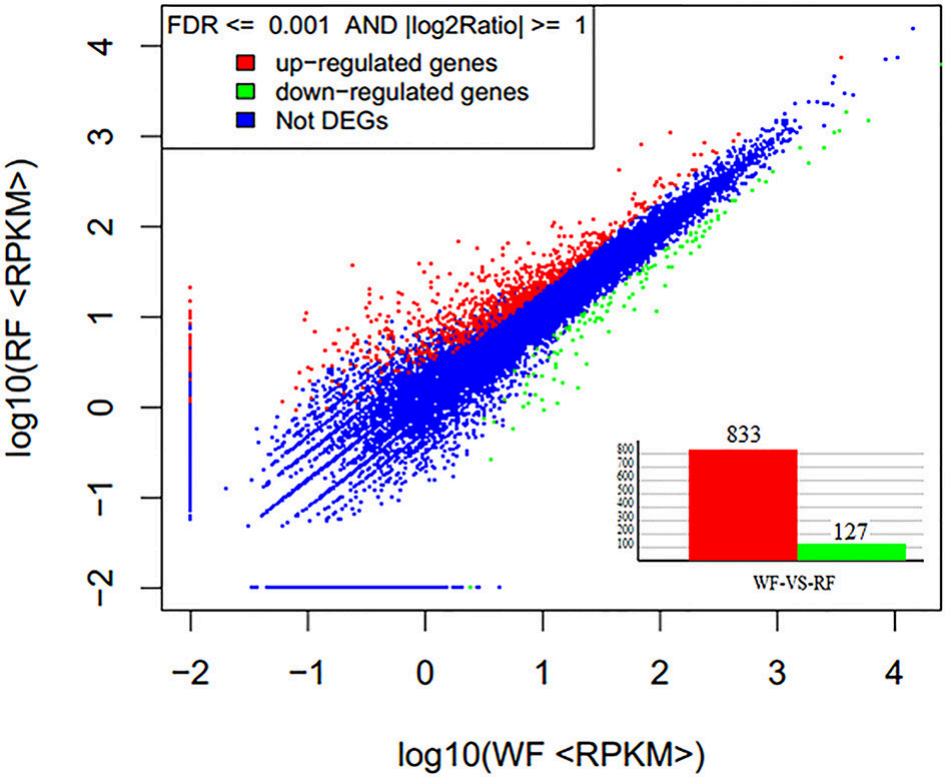
**Gene expression level of WF and RF flower of Japanese apricot**.

FDR ≤ 0.001 and log_2_ ratio (RF/WF) ≥ 1 were set as the threshold to estimate the significance of the gene expression difference between WF and RF. A total of 960 genes were identified differentially expressed, including 833 up-regulated and 127 down-regulated genes (Figure 7, Supplemental Table 3). Most of the DEG sets were up-regulated in the RF library. When the absolute value of log_2_ ratio (RF/WF) ≥ 5 in both WF and RF, 81 DEGs were up-regulated and only one gene was down-regulated. Variation in transcription level showed that significant difference between the two libraries.

### Detection of UFGT enzyme activity

**Figure 8:**
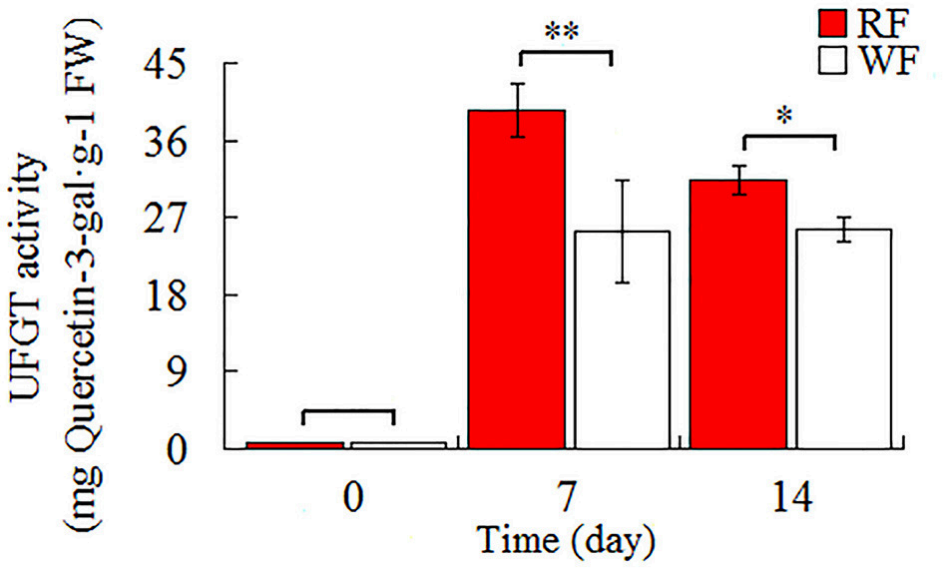
**Activities of UFGT in Japanese apricot flowers**. Different asterisks (^*^) indicate significant differences (^*^p < 0.05; ^**^p < 0.01).

As shown in Figure 3, the *UFGT* was significantly down-regulated in the WF consistent with RNA-seq and qRT-PCR result. In addition, *UFGT* is the last gene of anthocyanin pathway to regulate the key step for anthocyanin stability and water solubility in plant. Thus, we used the UPLC to determine the UFGT enzyme activity and the result was shown in Figure 8. The UPLC analysis showed one well-differentiated peak corresponding to quercetin-3-galactoside (Supplemental Figure S1). The UFGT enzyme activities were lower in the 0 day (initial stage), increased on 7 day (red stage), and the RF had a decreased at 14 day (balloon stage) while WF keep stable. When the color of petals was easily observed (7–day), the RF had a significantly higher enzyme activity than WF. The activity of UFGT is identical to the gene expression levels and accumulation of anthocyanins.

### Heterozygous point mutation be validated in the UFGT of japanese apricot flowers

**Figure 9:**
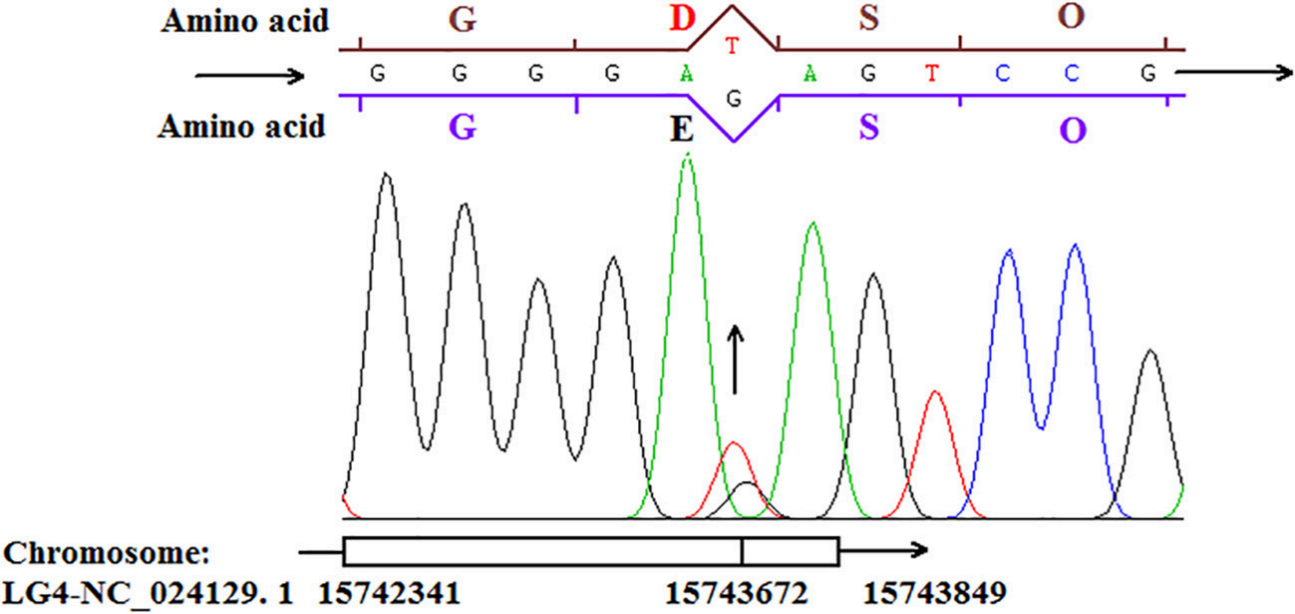
**The heterozygous point mutation of *UFGT* showing in Japanese apricot cv “Fuban Tiaozhi” flowers**.

The transcript levels of *UFGT*, corresponding to the anthocyanin levels, were higher in the red fruits than in the white-fleshed cultivar based on the study of the skin color in Japanese apricot (unpublished). In addition, several SNPs, which caused the nonsynonymous mutations, were identified in the white-fleshed cultivar compared to red cultivar. In our study, the primer pairs designed to amplify target sequence of *UFGT* were used to heterozygous point mutation validation. Within the amplified sequence, the heterozygous point mutation was validated in Japanese apricot flowers (Figure 9). The nonsynonymous mutation transform the T to K (T+G) caused the amino acid from D (GAT) to E (GAG) in UFGT. This mutation type occurred both in WF and RF while the pure white petals were nonexistent. Heterozygous point mutation would change the amino acid, and lead to the enzyme activity of UFGT deficiency. The activity of UFGT would be affected by unidentified factors to form either white or red flowers, but the mechanism of regulation need further study in Japanese apricot.

## Discussion

### Differentially expressed genes involved in flavonoids

Plant pigments that are perceived by humans to have color, have three main classes: flavonoids, carotenoids and betalains. Flavonoids (anthocyanins) play a crucial role in flower pigmentation and antioxidant activities (Nijveldt et al., 2001). Flavonoids are the main pigments in Japanese apricot flower petals. The content of anthocyanin showed that red petals had a higher level of anthocyanin than white petals (Figure 2). Therefore, identification of the DEGs related to flavonoid metabolism from different colored flowers is an essential step to elucidate mechanisms of variegated pigmentation. Transcriptome analysis of peach flowers relevant to variegation, four key structural genes involved in flavonoid biosynthesis were identified, including *CHS, C4H, F3H*, and *CHI* (Chen et al., 2014). In our study, by comparing RNA-seq libraries for red and white flowers, 53 differentially transcribed genes were found for three pathways involving flavonoids: flavonoid biosynthesis; anthocyanin biosynthesis; flavone and flavonol biosynthesis.

Flavonoids start from the phenylpropanoid pathway. Phenylalanine ammonia lyase (PAL) catalyses the conversion of phenylalanine to cinnamic acid, and 4-coumaroyl CoA ligase (4CL) activates 4-coumaric acid to 4-coumaroyl-CoA (Wang et al., 2011). The expression level of these genes may affect provision of substrates for biosynthesis of flavonoids. Naringenin is catalyzed by naringenin 3-dioxygenase (F3H, usually called flavanone 3-hydroxylase) to yield dihydrokaempferol (DHK), an important intermediate product. F3H also catalyzes the hydroxylation of flavanones to dihydroquercetin (DHQ) and dihydromyricetin (DHM), respectively. *F3H* and *C4H* are the key structural genes related to flavonoid biosynthesis. In *Petunia* and *Antirrhinum*, a mutation in the *f3h* locus caused a silent of F3H activity, resulting in white flowers (Schijlen et al., 2004). And by used antisense suppression to block the expression of *F3H*, the transgenic plants shown flower color modifications rang from attenuation to complete loss of reddish color (Zuker et al., 2002). In this study, we identified two *F3H* and one *C4H* that were up-regulated in the RF, which confirmed to the transcriptome results of peach flowers. Our results suggest that the low *C4H* and *F3H* expression levels in white petals could reduce DHK, DHQ and DHM formation, thereby inhibiting anthocyanin production. The low expression in WF is the reason for white petals in Japanese apricot.

Glutathione *S*-transferase (GST), as an anthocyanin carrier, could escort anthocyanins from the endoplasmic reticulum to the tonoplast (Sun et al., 2012). Insertion and excision of transposable elements (TEs) located in the promoter region of the *bz1* gene encoding *GST* causes unstable pigmentation in maize (Schiefelbein et al., 1988). TEs are also contributed to the unstable pigmentation phenotypic in ornamental plants, such as *carnation* (Nishizaki et al., 2011), *petunia* (Spelt et al., 2000). Three *GST* genes were found up-regulated in RF. Based on research in peach (Cheng et al., 2015), up-regulated *GST* cause the uneven accumulate and transport anthocyanins, finally formation variegation of flower petals.

### Genes involved in carotenoids

Carotenoids are plant pigments that play essential roles, such as photo-protective functions (Niyogi, 2000), and provision of substrates for biosynthesis of abscisic acid (Nambara and Marion-Poll, 2005) and other hormones (Auldridge et al., 2006). The important enzymes of carotenoid biosynthesis are phytoene desaturase (PDS), *zeta*-carotene desaturase (ZDS), and phytoene synthase (PSY). PDS catalyses phytoene to *zeta*-carotene, and ZDS converts *zeta*-carotene into lycopene (red color). Lycopene is an acyclic carotenoid in the polyene chain responsible for red color and antioxidant activity. In tomato pericarps, lycopene is the major contributor to red coloration (Gray et al., 1994). In our analysis, we found 20 DEGs involved in carotenoid biosynthesis. Among them, the gene ppa021185m, belonging to ZDS (EC: 1.3.5.6), showed a 9.2-fold up-regulation in RF. The expression of ZDS may determine the concentration of lycopene. Flavonoids (anthocyanins) are the main pigment in Japanese apricot, but the role of carotenoids needs further study.

### Some transcriptional factors related to anthocyanin accumulation

Anthocyanin accumulation in plants is controlled by transcriptional regulation of the genes encoding biosynthetic enzymes. Three classes of TFs, bHLH, MYB and WD40, that relate to flavonoid biosynthesis have been reported (Mano et al., 2007). TFs can act at different steps of the anthocyanin biosynthesis pathway (Huang et al., 2016). The plant MYB family, one of the most important families of transcription factors, is implicated in controlling plant-specific processes such as anthocyanin biosynthesis, signal transduction, environmental stress and disease resistance (Lewis et al., 2015). Anthocyanin-regulating MYBs have been isolated from many ornamental species, e.g., *Mimulus lewisii* (Sagawa et al., 2016) and *Petunia hybrida* (Shaipulah et al., 2015). In apple, MYBs that regulate the anthocyanin pathway genes have been examined (Ban et al., 2007). A MYB-related protein and a bHLH-containing protein have been shown to interact to activate genes in the anthocyanin pathway (Hatlestad et al., 2015). WD40 and bHLH proteins are involved in anthocyanin synthesis, like control the vacuolar pH in *petunia* flowers (Koes et al., 2005). In this study, we found 24 *MYB* genes, eight *bHLH* genes and two *WD40* genes (Supplemental Table 4), most of which were up-regulated in RF. We also found that bHLHs were all up-regulated in RF and ppa005343m was significantly expressed (log_2_ ratio >12) in RF which suggested that bHLH genes affected the expression levels of flavonoid biosynthesis-related genes although the exact regulatory mechanism remains unknown.

### Plant hormones as signaling molecules contribute to color variegation

Plant hormones have a crucial role in the regulation of flower development include the formation of pigments. Anthocyanin accumulation is enhanced by ABA, and suppressed by synthetic ET. ABA is a sesquiterpenoid (C15), and it can be synthesized by indirectly pathway in plants. In green plants, the specific steps of ABA biosynthesis occur in the plastids (Wilkinson and Burd, 2011). The ABA biosynthesis was catalyzed by zeaxanthin epoxidase (ZEP) and 9-*cis*-epoxycarotenoid dioxygenase (NCED) enzymes that product xanthoxin. The xanthoxin is then converted to ABA in the cytoplasm. NCEDs cleave the cis-isomers of violaxanthin and neoxanthin to a C15 product, xanthoxin, and a C25 metabolite. Biochemical and genetic evidences suggest that cleavage of 9-*cis*-xanthophylls is the key regulatory step in the ABA biosynthetic pathway (Priya and Siva, 2015). ABA has been proved that can induce anthocyanin biosynthesis (Kondo and Inoue, 1997). Kadomura-Ishikawa et al. (2015) reported that ABA independently regulated anthocyanin biosynthesis by activation of *FaMYB10* expression in *Fragaria* × *ananassa* fruit. In maize and tomato, *nced* mutants exhibit mild ABA-deficient phenotypes due to gene redundancy. In the water-stressed leaves, the accumulation of ABA was interrelated with an increased expression of *NCED* gene (Xiong and Zhu, 2003). Therefore, up-regulated *NCED* (ppa002804m) can increase the ABA biosynthesis and the ABA further could regulate the anthocyanin biosynthesis.

In α-linolenic acid metabolism, we found phospholipase A1, allene oxide cyclase (*AOC*), jasmonate *O*-methyltransferase and 12-oxophytodienoic acid reductase up-regulated in RF. The four genes took part in jasmonate biosynthesis. In the previous 2-DE, the protein abundance of AOC is higher in RF than WF (Zhou et al., 2015a). JAs are important cellular regulators involved in varying developmental processes (Wasternack and Hause, 2002) and activation of plant defense mechanisms (Cheong and Choi, 2003). MeJA are known to induce gene transcription directly, and promote the generation of ET by increase the synthesis of 1-aminocyclopropane-1-carboxylic acid (ACC) oxidase and/or its activity (Chang et al., 1997; Li et al., 2015).

ET is synthesized in two steps: from S-adenosyl-L-methionine to ACC and thence to ethylene (Kende, 1993). ACC synthase (ACS) plays an important role involved in the first step and ACC oxidase control the last step of ET biosynthesis. In this study, we found two genes (ppa004774m, ppa016458m) belonging to *ACS* and one gene (ppa014940m) belonging to ACC oxidase. These two *ACS* genes were significantly up-regulated by more than 7-fold while the ACC oxidaes was up-regulated about 3.5-fold, which may result in higher ET content in RF. ET is involved anthocyanin accumulation and floral color (Farzad et al., 2002; Villarreal et al., 2010). ET contribution to anthocyanin production was analyzed by observing the effect of exogenous ET and the ET inhibitors 1-MCP on floral color (Woltering and Somhorst, 1990). Anthocyanin biosynthesis was inhibited by specific inhibitor of ET receptors in the berry skin (Blankenship and Dole, 2003). Pigment biosynthesis may result from ET production. Ethylene-responsive transcription factor 1 (ERF1) is a transcriptional cascade to ET signaling (Alexander and Grierson, 2002). We found 3 DEGs belong to *ERF1*, which directly activates transcription of ethylene-responsive pathogenesis-related genes.

### UFGT is the key gene for red color in *P. mume*

*UFGT* is the last gene in the anthocyanin pathway (Chen et al., 2015). The UFGT, as the major control point for anthocyanin production, transfers the glucose to the C-3 hydroxyl group of anthocyanidins which resulting in the colored pigments of anthocyanins 3-*O*-glucosides (Zhao et al., 2012). It is the key step for anthocyanin stability and water solubility in plant (Yoshihara et al., 2005). The expression of the *UFGT* was under developmental control as well as affected by plant hormones ET and ABA (Chervin et al., 2009; Chen et al., 2015). Up-regulation of *UFGT* is important for generation of colored pigments and stabilizing anthocyanins, and contributed to accumulation of water-soluble pigment in the vacuoles. In the grapes, the expression analysis of *UFGT* genes in white and red-skinned reve*al*ed that the *UFGT* was present, but not expressed in the white skins (Kobayashi et al., 2001), also exist in apples (Meng et al., 2015), and tobacco flowers (Liao et al., 2015). The loss of function or low expression of *UFGT* leads to loss or reduced accumulation of anthocyanin (Kubo et al., 2007). The lowest expression level of *NtUFGT* was produced almost-white flowers with the lowest anthocyanin content in transgenic tobacco (Zhu et al., 2015). Thus, expression of the *UFGT* coincides with anthocyanin accumulation. Chervin et al. (2009) ran transient expression analyses shown that ABA, sugar and ET can all stimulate expression from the *UFGT* promoter.

In anthocyanin biosynthesis, *UFGT* was found up-regulated in the RF library. It is benefit for the RF to accumulate more pigments to show the red color. For further analysis of the UFGT enzyme activity between RF and WF, we used the UPLC to quantification of enzyme activity. The results suggest that in initial stage without redness color the enzyme activity of UFGT is low. And the enzyme activity of UFGT was quickly improving during the anthocyanin biosynthesis. The RF had a higher enzyme activity of UFGT than WF when petals color observed differences. The result of enzyme activity is accordance with the RNA-seq. Based on the nonsynonymous mutation validation, which caused the heterozygous point mutation were occurred in the *UFGT* in the cv “Fuban Tiaozhi” Mei petals. The nonsynonymous mutation may affect the activity of enzyme. The mutation occurred in both two samples because of the two color is coexisted in the petals. We suspect that only heterozygote of the SNPs might show variegation in flowers. When the mutated cells distributed within the different layers, the different degree and position of white will model the variegated coloration in flowers. So it is speculated that the UFGT enzyme might have the key role in the color regulation. But the mechanism underlying the mutation formation in Japanese apricot requires further study.

### A model of floral color variegation hypothesized in japanese apricot

**Figure 10:**
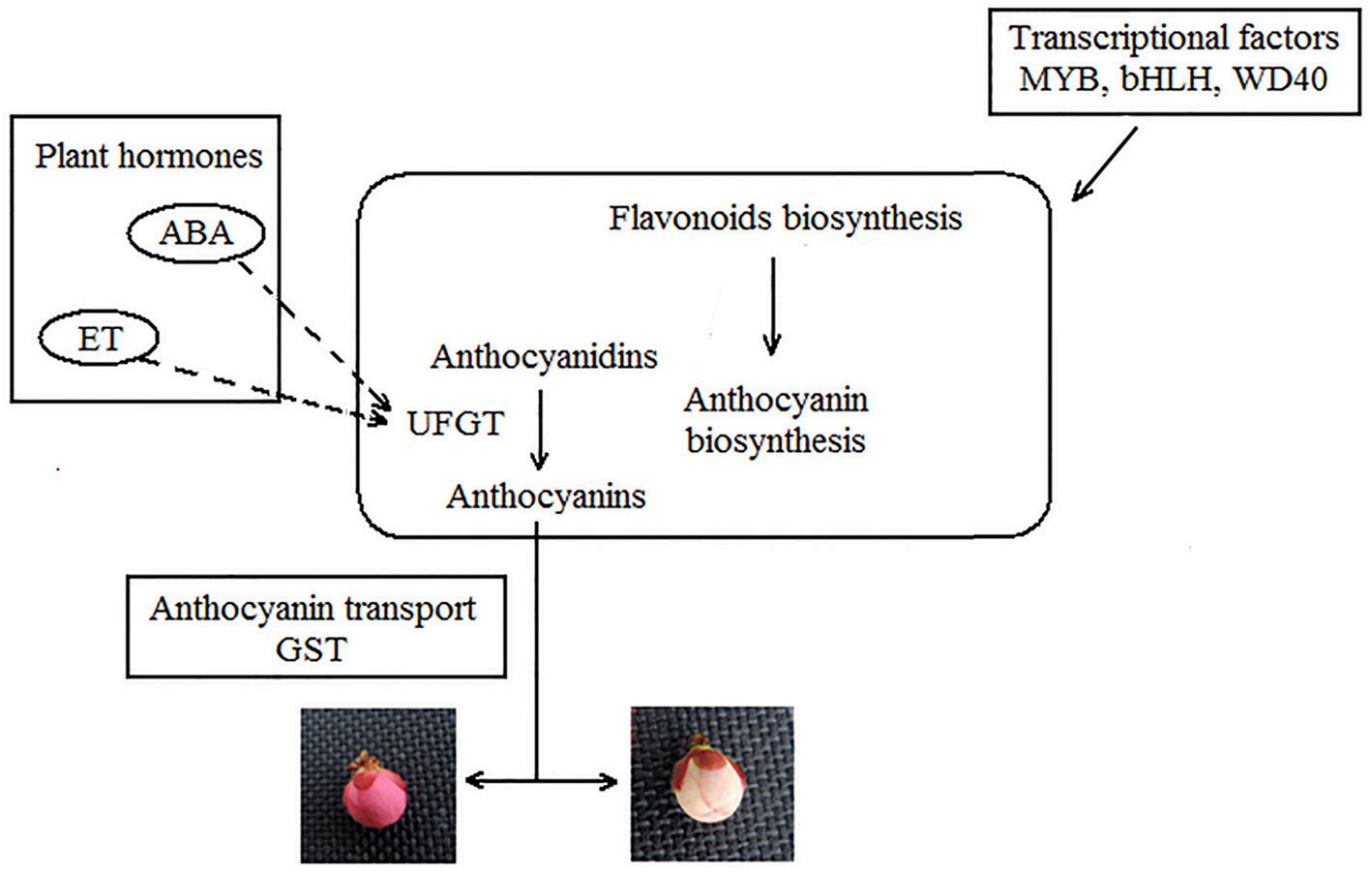
**Model of Japanese apricot floral color variegation**.

According to the results of RNA-seq and other research, a model of Japanese apricot floral color variegation could be preliminary as shown in Figure 10. We suspect that, the unknown stress is probably the external cause inducing floral color variegation. It is notable that transcriptional factors and plant hormones may play a key role in modification involved in gene regulation, transposon silencing (Jeong et al., 2004; Zhu et al., 2015). Plant hormones, like ET and ABA induce structural gene *UFGT* expression, as signaling molecules contribute to color variegation (Chervin et al., 2009; Chen et al., 2015). Plant pigments biosynthesis is the key step for floral color. We found that 53 DEGs were involved in flavonoid biosynthesis. The high level expression and enzyme activity of UFGT may determine the concentration of anthocyanins and contribute to anthocyanin accumulation. Accumulation more stabilizing anthocyanins is benefit to red color. The nonsynonymous mutation in the *UFGT* can affect the activity of enzyme. The higher gene expression and enzyme activity of UFGT contributes to stable anthocyanin production endow petals with red color, while the white color be observed under lower enzyme activity. Then the anthocyanin transporter GST transports the anthocyanin. While the UFGT enzyme and pigment uneven distribution lead to variegation in Japanese apricot buds.

## Conclusions

Although the pathways of pigments biosynthesis are increasing being determined, few studies have shown that the molecular mechanisms of variegation in Japanese apricot. In this study, the transcriptome provided comprehensive sequence profiling data for flower color in Japanese apricot cv “Fuban Tiaozhi” Mei. A total of 960 DEGs were identified in WF vs. RF samples. Pathway enrichment annotation revealed that highly ranked genes were involved in flavonoid biosynthesis, anthocyanin biosynthesis. Among them, the *UFGT* is located in the key step for anthocyanin stability and water solubility, and shown significantly up-regulated in the RF by the RNA-seq analysis and qRT-PCR assay. The high level expression and enzyme activity of UFGT may determine the concentration of anthocyanins and accumulation more stabilizing anthocyanin which endow petals with red color. Conversely, the white color would be observed under lower enzyme activity in petals. Our results provide valuable molecular information on the formation of flower color variegation in Japanese apricot. It should promote further investigations into the detailed regulatory pathways regulating pigments biosynthesis and contribute to a better understanding of the formation of flower color variegation in Japanese apricot.

## Author contributions

ZG conceived and designed the study. XW performed the experiments, analyzed the data, and wrote the manuscript. XN and YZ helped to perform the experiments. QG assisted with data analysis and performed the experiments.

### Conflict of interest statement

The authors declare that the research was conducted in the absence of any commercial or financial relationships that could be construed as a potential conflict of interest.
