# Supplementary material for: UFGT: The Key Enzyme Associated with the Petals Variegation in Japanese Apricot
Source: Front Plant Sci. 2017 Feb 7;8:108. doi: 10.3389/fpls.2017.00108 (PMC5293763; doi:10.3389/fpls.2017.00108)
Supplement: Supplementary file 1 [file Table1.DOC]

Supplemental Table 1 Primers used in real-time quantitative PCR (RT-PCR).

| *Gene* | *Primer Name* | **Sequence (5’-3’)** |
| --- | --- | --- |
| ppa016929m | Forward | CCTTCAATACCAACACTTCC |
|  | Reverse | CAATGGAGGCAGAGTAGT |
| ppa025637m | Forward | CTCTGCTCTTCTTGGTCTG |
|  | Reverse | AGATTCCTCCATCAACTCAG |
| ppa024319m | Forward | CTTCTCCCACAACTCCAT |
|  | Reverse | GGCGACGAACCATTATTG |
| ppa025302m | Forward | CGATTGCTTATGAGGTCAAG |
|  | Reverse | GGTTCTCCAAGAGGTAGG |
| ppa016488m | Forward | GCCAGATAACAACACCATAC |
|  | Reverse | CCTTGAAGTAGCACAACAC |
| ppa022833m | Forward | TCTGCTCTTCTTGGTCTG |
|  | Reverse | AAGATTCCTCCATCAACTCA |
| ppa012795m | Forward | GTGAGGATGTGAATGAGTTC |
|  | Reverse | TGGTGAGCATAATACAAGGA |
| ppa1027213m | Forward | CCACCAACTCTGTTCTCT |
|  | Reverse | CTGCCAATCCAATCTGAAG |
| ppa007719m | Forward | TGGTGGTGAGGATATTGG |
|  | Reverse | GCTCTTGTATGTTGCGTTA |
| ppa008269m | Forward | TGAGCAAGGACAGCATAT |
|  | Reverse | TCGCCAAGGTTAATGACA |
| ppa012260m | Forward | CTCCTTCTTCACCACTTCTA |
|  | Reverse | CAAACACCTGACCTTCAAA |
| UFGT | Forward | ATTAAATGCCTTTGAATTGGTA |
|  | Reverse | CAAAATCAGAACCACCGAAT |
| Actin | Forward | TGAAGCATACACCTATGATGATGAAG |
|  | Reverse | CTTTGACAGCACCAGTAGATTCC |
