# Supplementary material for: UFGT: The Key Enzyme Associated with the Petals Variegation in Japanese Apricot
Source: Front Plant Sci. 2017 Feb 7;8:108. doi: 10.3389/fpls.2017.00108 (PMC5293763; doi:10.3389/fpls.2017.00108)
Supplement: Supplementary file 2 [file Table2.DOC]

Supplemental Table 2. Quality assessment of reads for two RNA-Seq libraries.

|  | *Composition of Raw Reads (RF)* | **Composition of Raw Reads (WF)** |
| --- | --- | --- |
| Containing Adaptor | 76769 (0.65%) | 84092 (0.70%) |
| Containing N | 395 (0.00%) | 411 (0.00%) |
| Low Quality | 9612 (0.08%) | 7483 (0.06%) |
| Clean Reads | 11669841 (99.26%) | 11983115 (99.24%) |
