# Supplementary material for: UFGT: The Key Enzyme Associated with the Petals Variegation in Japanese Apricot
Source: Front Plant Sci. 2017 Feb 7;8:108. doi: 10.3389/fpls.2017.00108 (PMC5293763; doi:10.3389/fpls.2017.00108)
Supplement: Supplementary file 4 [file Table4.DOC]

Supplemental Table 4 Differentially expressed of TFs.

| *Gene ID* | *WF-RPKM* | *RF-RPKM* | *Log2 Ratio (RF/WF)* |
| --- | --- | --- | --- |
| MYB-like DNA-binding domains | | | |
| ppa026553m | 0.18 | 7.31 | 5.31 |
| ppa021577m | 0.90 | 12.30 | 3.78 |
| ppa022205m | 0.52 | 6.36 | 3.60 |
| ppa008877m | 1.59 | 13.03 | 3.03 |
| ppa021568m | 1.24 | 9.81 | 2.99 |
| ppa007438m | 0.84 | 6.43 | 2.94 |
| ppa011549m | 0.98 | 7.02 | 2.83 |
| ppa023768m | 6.36 | 44.96 | 2.82 |
| ppa009439m | 13.54 | 52.35 | 1.95 |
| ppa014060m | 4.61 | 17.28 | 1.91 |
| ppa014599m | 1.82 | 6.52 | 1.84 |
| ppa015954m | 2.77 | 9.81 | 1.82 |
| ppa007594m | 12.17 | 41.03 | 1.75 |
| ppa007753m | 7.56 | 23.85 | 1.66 |
| ppa009143m | 11.64 | 36.64 | 1.65 |
| ppa008450m | 12.31 | 32.69 | 1.41 |
| ppa008539m | 5.79 | 13.27 | 1.20 |
| ppa006769m | 61.16 | 131.65 | 1.11 |
| ppa004107m | 49.35 | 12.92 | -1.93 |
| ppa006715m | 77.19 | 30.49 | -1.34 |
| ppa023366m | 14.07 | 6.58 | -1.10 |
| ppa011751m | 410.55 | 192.17 | -1.10 |
| ppa001765m | 216.77 | 102.84 | -1.08 |
| ppa023154m | 21.50 | 10.61 | -1.02 |
| Basic helix-loop-helix DNA-binding domain | | | |
| ppa005343m | 0.01 | 2.74 | 8.10 |
| ppa017791m | 0.22 | 7.63 | 5.12 |
| ppa019406m | 2.02 | 8.69 | 2.11 |
| ppa021226m | 9.89 | 31.67 | 1.68 |
| ppa010134m | 3.73 | 11.51 | 1.63 |
| ppa017228m | 3.86 | 9.54 | 1.31 |
| ppa007656m | 3.64 | 8.84 | 1.28 |
| ppa005254m | 9.54 | 22.94 | 1.27 |
| WD40 | | | |
| ppa021636m | 9.084349 | 18.9575 | 1.061313713 |
| ppa017540m | 28.42468 | 9.795323 | -1.536979118 |
