# Supplementary figures and images for: UFGT: The Key Enzyme Associated with the Petals Variegation in Japanese Apricot
Source: Front Plant Sci. 2017 Feb 7;8:108. doi: 10.3389/fpls.2017.00108 (PMC5293763; doi:10.3389/fpls.2017.00108)

Supplemental Figure 1. The UPLC analysis showed peak corresponding to quercetin-3-galactoside.

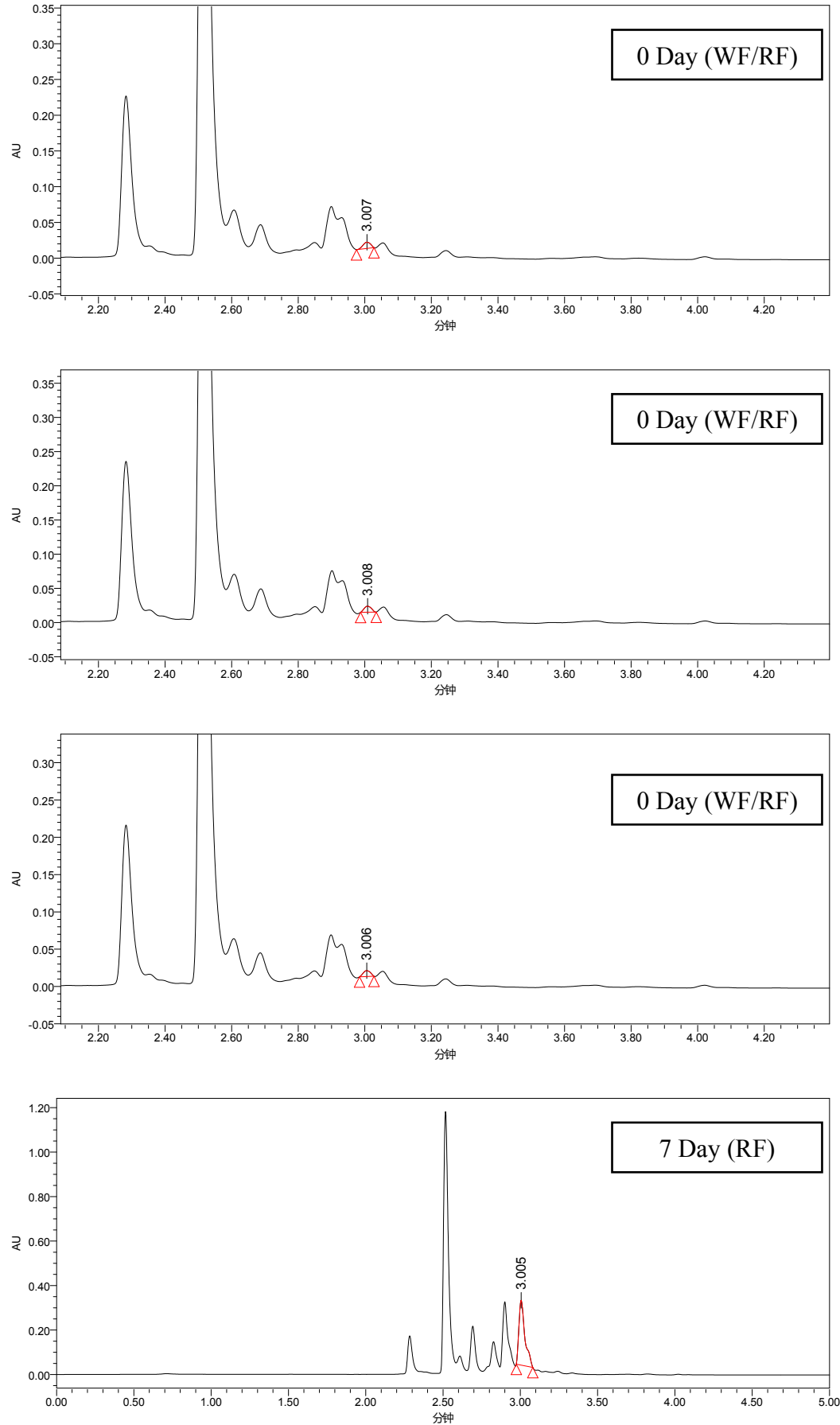

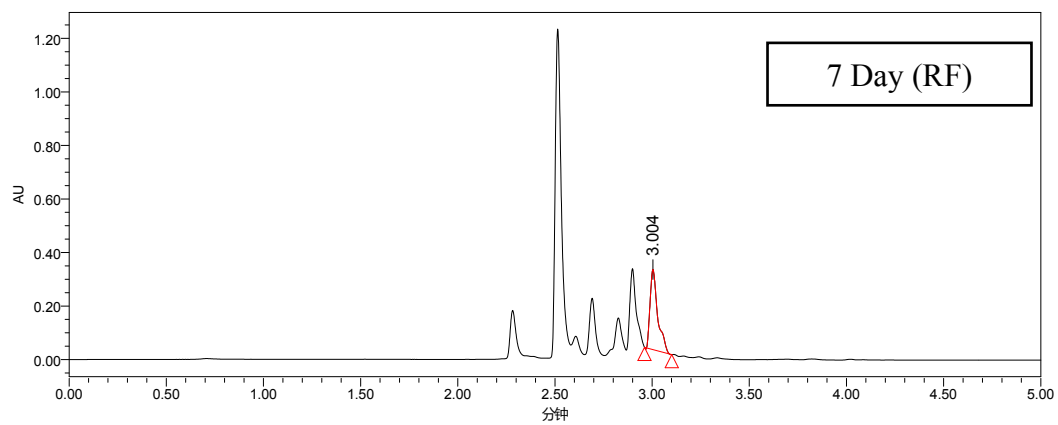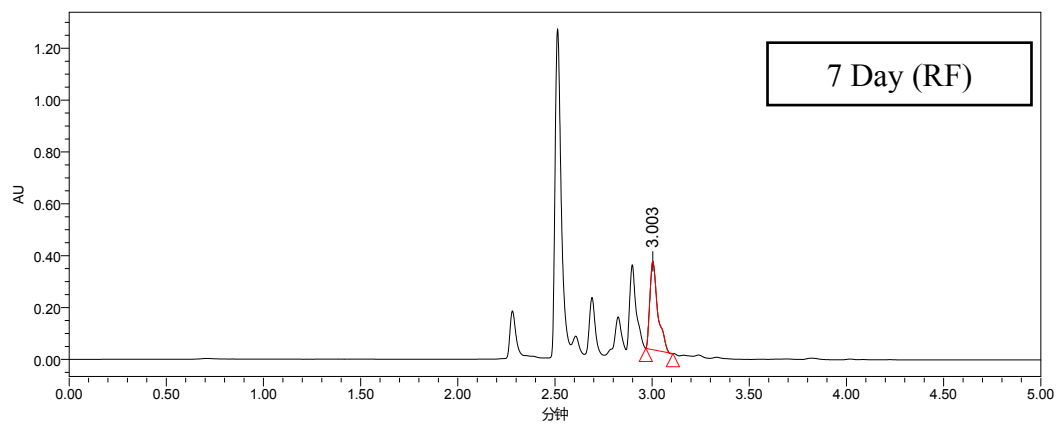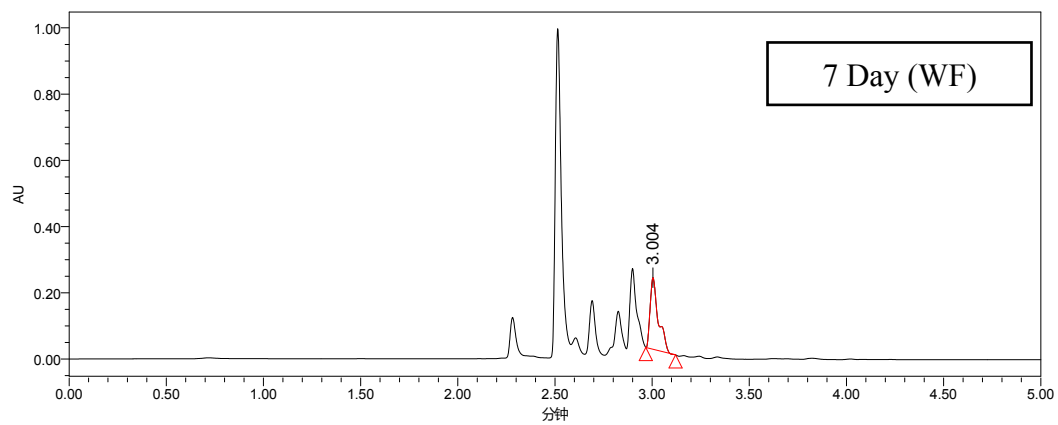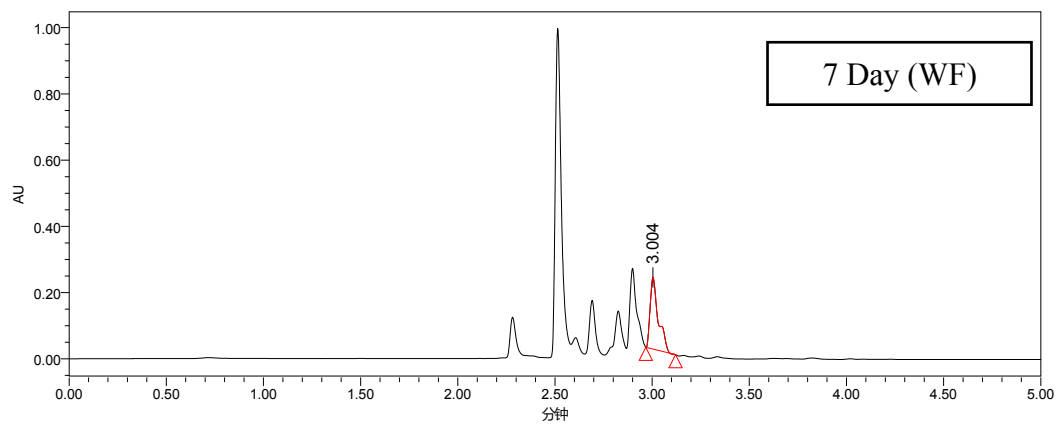

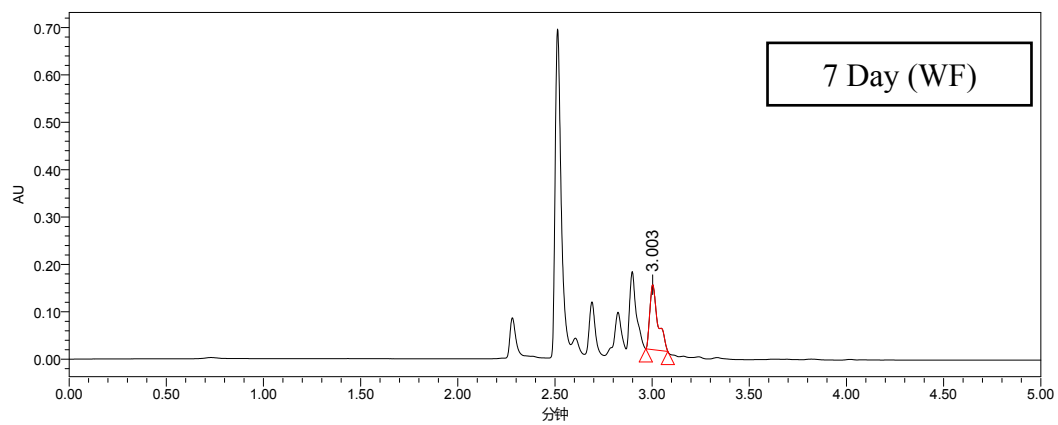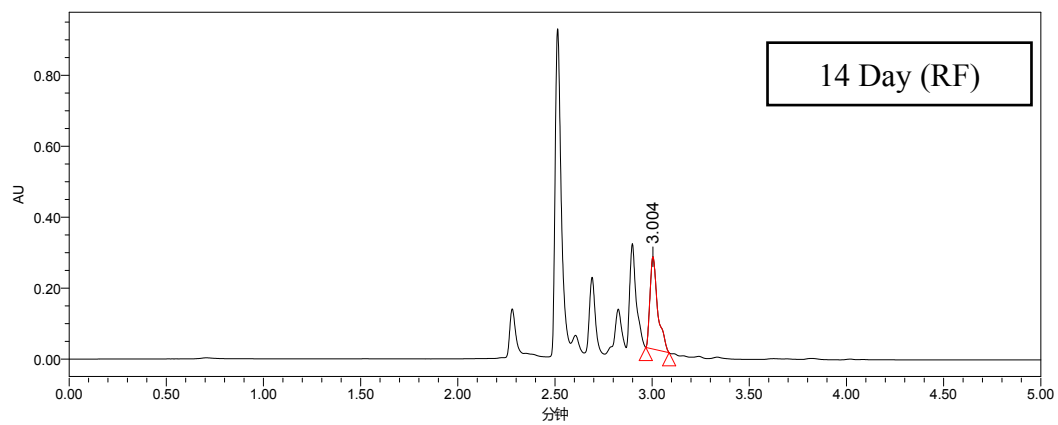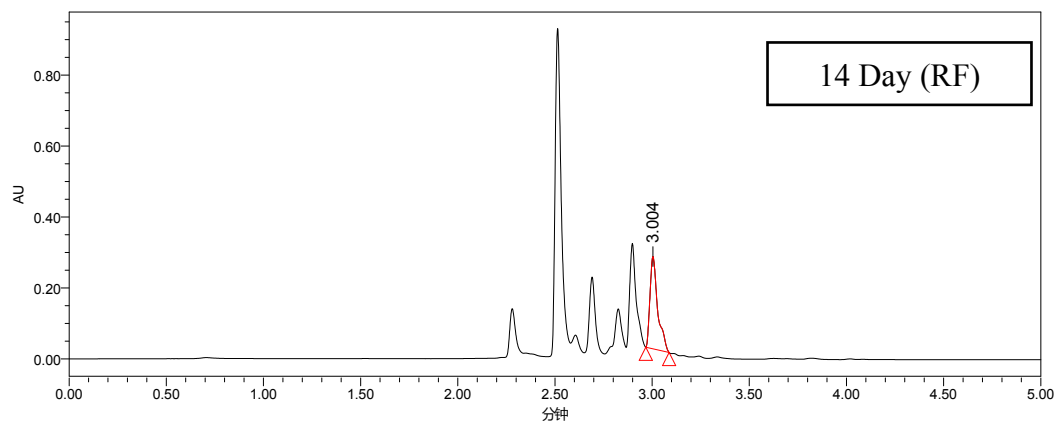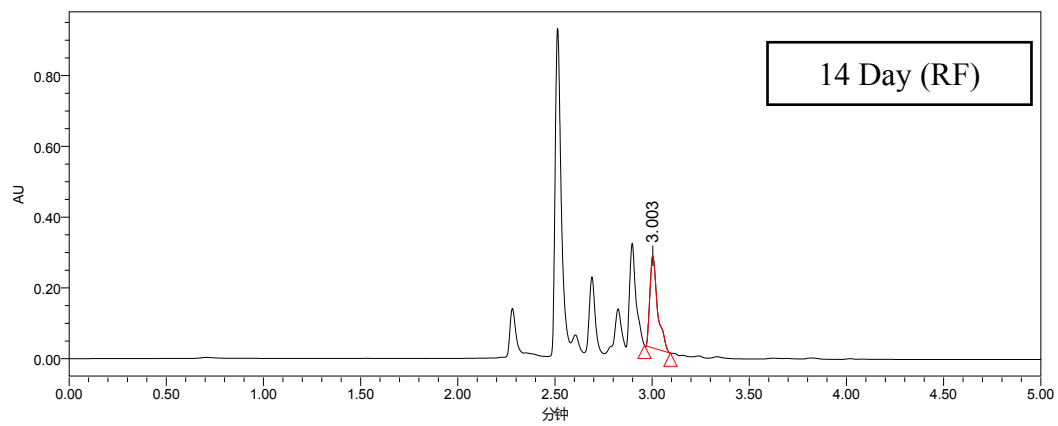

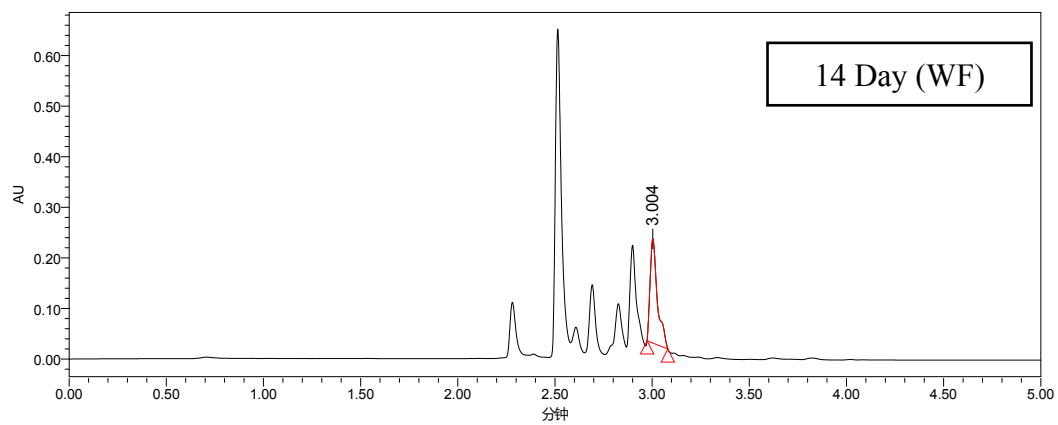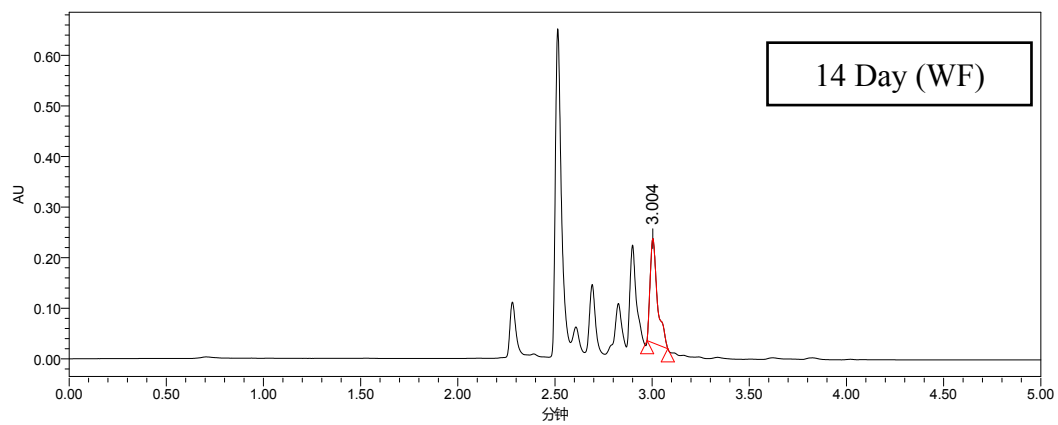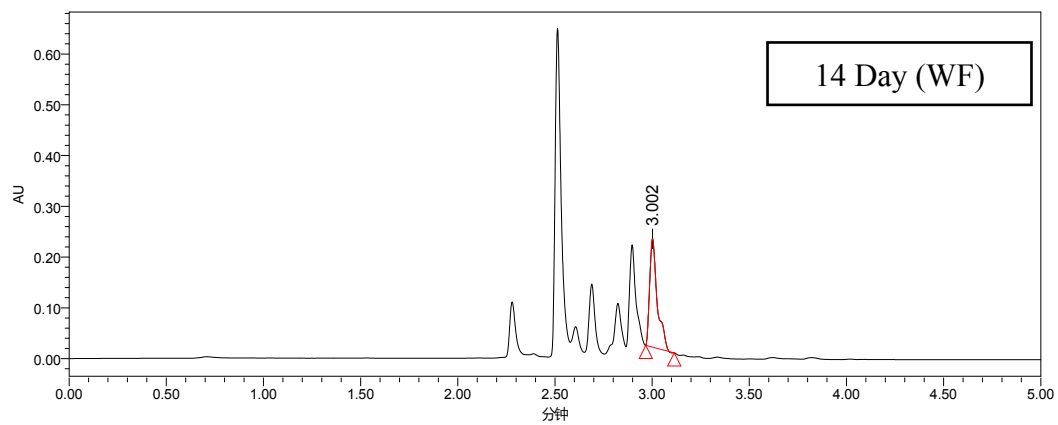

Supplement: Supplementary file 5 [file Image1.PDF]
